# Supplementary material for: Physical vitrification and nanowarming at liter-scale CPA volumes: toward organ cryopreservation
Source: Nat Commun. 2025 Sep 26;16:8511. doi: 10.1038/s41467-025-63483-2 (PMC12474906; doi:10.1038/s41467-025-63483-2)
Supplement: Supplementary file 2 — Reporting Summary [file 41467_2025_63483_MOESM2_ESM.pdf]

nature portfolio

Corresponding author(s): Erik B. Finger and John C. Bischof

Last updated by author(s): Aug 1, 2025

Reporting Summary

Nature Portfolio wishes to improve the reproducibility of the work that we publish. This form provides structure for consistency and transparency in reporting. For further information on Nature Portfolio policies, see our Editorial Policies and the Editorial Policy Checklist.

Statistics

For all statistical analyses, confirm that the following items are present in the figure legend, table legend, main text, or Methods section.

n/a	Confirmed	
☐	☒	The exact sample size (*n*) for each experimental group/condition, given as a discrete number and unit of measurement
☐	☒	A statement on whether measurements were taken from distinct samples or whether the same sample was measured repeatedly
☒	☐	The statistical test(s) used AND whether they are one- or two-sided<br>*Only common tests should be described solely by name; describe more complex techniques in the Methods section.*
☒	☐	A description of all covariates tested
☒	☐	A description of any assumptions or corrections, such as tests of normality and adjustment for multiple comparisons
☒	☐	A full description of the statistical parameters including central tendency (e.g. means) or other basic estimates (e.g. regression coefficient) AND variation (e.g. standard deviation) or associated estimates of uncertainty (e.g. confidence intervals)
☒	☐	For null hypothesis testing, the test statistic (e.g. *F*, *t*, *r*) with confidence intervals, effect sizes, degrees of freedom and *P* value noted<br>*Give P values as exact values whenever suitable.*
☒	☐	For Bayesian analysis, information on the choice of priors and Markov chain Monte Carlo settings
☒	☐	For hierarchical and complex designs, identification of the appropriate level for tests and full reporting of outcomes
☒	☐	Estimates of effect sizes (e.g. Cohen's *d*, Pearson's *r*), indicating how they were calculated

Our web collection on statistics for biologists contains articles on many of the points above.

Software and code

Policy information about availability of computer code

Data collection	Commercial software used in this study were Qualitrol Neoptix Optilink, Micronor FOTEMP ASSISTANT, COMSOL 5.4, Altair Flux 2D
Data analysis	Graphpad Prism 10, Microsoft Office 365 Excel, NIKON 3D CT Pro, Volume Graphics VGstudio Max 3.2

For manuscripts utilizing custom algorithms or software that are central to the research but not yet described in published literature, software must be made available to editors and reviewers. We strongly encourage code deposition in a community repository (e.g. GitHub). See the Nature Portfolio guidelines for submitting code & software for further information.

Data

Policy information about availability of data

All manuscripts must include a data availability statement. This statement should provide the following information, where applicable:

- Accession codes, unique identifiers, or web links for publicly available datasets
- A description of any restrictions on data availability
- For clinical datasets or third party data, please ensure that the statement adheres to our policy

Raw data for each figure can be found in supplementary information

Research involving human participants, their data, or biological material

Policy information about studies with human participants or human data. See also policy information about sex, gender (identity/presentation), and sexual orientation and race, ethnicity and racism.

Reporting on sex and gender	*Use the terms sex (biological attribute) and gender (shaped by social and cultural circumstances) carefully in order to avoid confusing both terms. Indicate if findings apply to only one sex or gender; describe whether sex and gender were considered in study design; whether sex and/or gender was determined based on self-reporting or assigned and methods used. Provide in the source data disaggregated sex and gender data, where this information has been collected, and if consent has been obtained for sharing of individual-level data; provide overall numbers in this Reporting Summary. Please state if this information has not been collected. Report sex- and gender-based analyses where performed, justify reasons for lack of sex- and gender-based analysis.*
Reporting on race, ethnicity, or other socially relevant groupings	*Please specify the socially constructed or socially relevant categorization variable(s) used in your manuscript and explain why they were used. Please note that such variables should not be used as proxies for other socially constructed/relevant variables (for example, race or ethnicity should not be used as a proxy for socioeconomic status). Provide clear definitions of the relevant terms used, how they were provided (by the participants/respondents, the researchers, or third parties), and the method(s) used to classify people into the different categories (e.g. self-report, census or administrative data, social media data, etc.) Please provide details about how you controlled for confounding variables in your analyses.*
Population characteristics	*Describe the covariate-relevant population characteristics of the human research participants (e.g. age, genotypic information, past and current diagnosis and treatment categories). If you filled out the behavioural & social sciences study design questions and have nothing to add here, write "See above."*
Recruitment	*Describe how participants were recruited. Outline any potential self-selection bias or other biases that may be present and how these are likely to impact results.*
Ethics oversight	*Identify the organization(s) that approved the study protocol.*

Note that full information on the approval of the study protocol must also be provided in the manuscript.

Field-specific reporting

Please select the one below that is the best fit for your research. If you are not sure, read the appropriate sections before making your selection.

☒ Life sciences ☐ Behavioural & social sciences ☐ Ecological, evolutionary & environmental sciences

For a reference copy of the document with all sections, see nature.com/documents/nr-reporting-summary-flat.pdf

Life sciences study design

All studies must disclose on these points even when the disclosure is negative.

Sample size	We performed at least 3 replicates (i.e. independent experiments) for each experimental condition, which is sufficient and commonly used to report physical results based on the literature. A total of n=5 livers were used based upon the availability of donor animals (i.e. pigs).
Data exclusions	All porcine livers that underwent the protocol without incident were included. Two livers experiencing technical failures and were excluded. One experienced incomplete perfusion due to kinked hose whereas the other was placed incorrectly in the CRF thereby maximizing instead of minimizing the characteristic length (Lc) which led to cooling failure.
Replication	Replication were performed on different days with distinct liver samples. Total of ≥3 replicates were performed for each liver and CPA experiment. It is to be noted that microCT was not repeated on all the livers due to the fact that we needed to break the liver into smaller pieces to fit in the CT machine.<br><br>To enable reproducibility by other labs, we have specifically listed the control rate freezer (CRF) vitrification protocols in the Method section as Table 1 and Fig. S7 and the liver perfusion protocol in supplementary as Fig. S5. For CPA and nanoparticle preparation we have cited the literature which describes the step by step protocols for each in the methods section. Furthermore, Table S2 lists the detailed composition of CPAs used in the study.
Randomization	Livers were taken from animals selected randomly between 9-30kg (n=3 ~25-30kg, n=2 ~9kg). The smaller livers (and animals) were the result of animal vendor constraints during the response time window. The larger livers came from pigs used for concurrent studies at the University of Minnesota.
Blinding	Blinding was not possible for this experimental design. Livers were utilized when they became available therefore obviating a single blind methodology.

Reporting for specific materials, systems and methods

We require information from authors about some types of materials, experimental systems and methods used in many studies. Here, indicate whether each material, system or method listed is relevant to your study. If you are not sure if a list item applies to your research, read the appropriate section before selecting a response.

Materials & experimental systems

n/a	Involved in the study
☒	☐ Antibodies
☒	☐ Eukaryotic cell lines
☒	☐ Palaeontology and archaeology
☐	☒ Animals and other organisms
☒	☐ Clinical data
☒	☐ Dual use research of concern
☒	☐ Plants

Methods

n/a	Involved in the study
☒	☐ ChIP-seq
☒	☐ Flow cytometry
☒	☐ MRI-based neuroimaging

Animals and other research organisms

Policy information about studies involving animals; ARRIVE guidelines recommended for reporting animal research, and Sex and Gender in Research

Laboratory animals	9-30kg (n=3 ~25-30kg, n=2 ~9kg) male Yorkshire pigs sourced from local vendors (Midwest Research Swine, Premium BioSource and MohrVet, MN).
Wild animals	The study did not involve wild animals.
Reporting on sex	Vitrification experiments were performed using livers from male pigs. Male livers were selected for their larger size and therefore lower expected cooling rates which makes vitrification more challenging (i.e. limiting case). Female livers are typically smaller and hence would be expected to vitrify using a protocol that was successful for larger male livers.
Field-collected samples	The study did not involve samples collected from field.
Ethics oversight	The Institutional IACUC committee from the University of Minnesota (protocol #2111A39564) approved all animal studies.

Note that full information on the approval of the study protocol must also be provided in the manuscript.

Plants

Seed stocks	N/A
Novel plant genotypes	N/A
Authentication	N/A
